# The age of blood in pediatric intensive care units (ABC PICU): study protocol for a randomized controlled trial

**DOI:** 10.1186/s13063-018-2809-y

**Published:** 2018-07-28

**Authors:** Marisa Tucci, Jacques Lacroix, Dean Fergusson, Allan Doctor, Paul Hébert, Robert A. Berg, Jaime Caro, Cassandra D. Josephson, Stéphane Leteurtre, Kusum Menon, Kenneth Schechtman, Marie E. Steiner, Alexis F. Turgeon, Lucy Clayton, Tina Bockelmann, Philip C. Spinella, for the Canadian Critical Care Trials Group, the Pediatric Critical Care Blood Research Network (BloodNet), the Pediatric Acute Lung Injury and Sepsis Investigators (PALISI) Network

**Affiliations:** 1 From the Division of Pediatric Critical Care Medicine, Department of Pediatrics, Sainte-Justine Hospital, Faculty of Medicine, Université de Montréal, Montréal, QC Canada; 2 Clinical Epidemiology Program, Ottawa Hospital Research Institute and Faculty of Medicine, University of Ottawa, Ottawa, ON Canada; 3 Division of Critical Care, Department of Pediatrics, Washington University in St. Louis, St. Louis, MO USA; 4 Division of Critical Care Medicine, Department of Medicine, Montreal University Health Center, Faculty of Medicine, Université de Montréal, Montréal, QC Canada; 5 Division of Pediatric Critical Care Medicine, Department of Anesthesiology and Critical Care Medicine, Children’s Hospital of Philadelphia, Faculty of Medicine, University of Pennsylvania, Philadelphia, PA USA; 6 Department of Epidemiology, Biostatistics and Occupational Health, Faculty of Medicine, McGill University, Montreal, QC Canada; 7 Evidera, Boston, MA USA; 8 Departments of Pathology and Pediatrics, Emory University School of Medicine and Children’s Healthcare of Atlanta, Atlanta, GA USA; 9 University of Lille, CHU Lille, EA 2694 – Santé Publique : épidémiologie et qualité des soins, F-59000 Lille, France; 10 Division of Pediatric Critical Care Medicine, Department of Pediatrics, Children’s Hospital of Eastern Ontario, Faculty of Medicine, University of Ottawa, Ottawa, ON Canada; 11 Clinical Epidemiology Program, St. Louis Children’s Hospital, Faculty of Medicine, Washington University School of Medicine, St. Louis, MO USA; 12 Division of Pediatric Hematology-Oncology and Division of Pulmonary and Critical Care, Department of Pediatrics, University of Minnesota, Minneapolis, MN USA; 13 Division of Critical Care Medicine, Department of Anesthesiology and Critical Care Medicine, and CHU de Québec-Université Laval Research Centre, Population Health and Optimal Health Practices Unit, Université Laval, Québec City, QC Canada; 14 From the Clinical Research Unit, Research Center, Sainte-Justine Hospital, Université de Montréal, Montréal, QC Canada; 15 Sainte-Justine Hospital, 3175 Côte Sainte-Catherine, Montréal, QC H3T 1C5 Canada

**Keywords:** Blood, Children, Critical care, Erythrocyte, Red blood cell (RBC), Intensive care, Mortality, Randomized controlled trial, Study protocol, Transfusion

## Abstract

**Background:**

The “Age of Blood in Children in Pediatric Intensive Care Unit” (ABC PICU) study is a randomized controlled trial (RCT) that aims to determine if red blood cell (RBC) unit storage age affects outcomes in critically ill children. While RBCs can be stored for up to 42 days in additive solutions, their efficacy and safety after long-term storage have been challenged. Preclinical and clinical observational evidence suggests loss of efficacy and lack of safety of older RBC units, especially in more vulnerable populations such as critically ill children. Because there is a belief that shorter storage will improve outcomes, some physicians and institutions systematically transfuse fresh RBCs to children. Conversely, the standard practice of blood banks is to deliver the oldest available RBC unit (first-in, first-out policy) in order to decrease wastage.

**Methods/design:**

The ABC PICU study, is a double-blind superiority trial comparing the development of “New or Progressive Multiple Organ Dysfunction Syndrome” (NPMODS) in 1538 critically ill children randomized to either transfusion with RBCs stored for ≤ 7 days or to standard-issue RBCs (oldest in inventory). Patients are being recruited from 52 centers in the US, Canada, France, Italy, and Israel.

**Discussion:**

The ABC PICU study should have significant implications for blood procurement services. A relative risk reduction of 33% is postulated in the short-storage arm. If a difference is found, this will indicate that fresher RBCs do improve outcomes in the pediatric intensive care unit population and would justify that use in critically ill children.

If no difference is found, this will reassure clinicians and transfusion medicine specialists regarding the safety of the current system of allocating the oldest RBC unit in inventory and will discourage clinicians from preferentially requesting fresher blood for critically ill children.

**Trial registration:**

ClinicalTrials.gov, ID: NCT01977547. Registered on 6 November 2013.

**Electronic supplementary material:**

The online version of this article (10.1186/s13063-018-2809-y) contains supplementary material, which is available to authorized users.

## Background

Red blood cells (RBCs) are transfused in anemic patients primarily to maintain or to improve oxygen (O_2_) delivery and consumption by vital organs and, therefore, prevent or reverse O_2_ debt, which may result in shock and/or multiple organ dysfunction syndrome (MODS). Standard policy for the majority of children in North American and European hospitals is to dispense the oldest RBC unit available in the blood bank [1, 2]. While this approach limits wastage, the impact upon transfusion efficacy and safety, specifically in critically ill populations, is a concern due to immune modulation, decreased RBC deformability, altered nitric oxide metabolism, and increased coagulation [3–5]. Regulatory agencies have established the upper limit of RBC storage based upon mean hemolysis of less than 1% (0.8% in Europe) and > 75% of transfused circulating RBCs still viable in healthy volunteers 24 h after transfusion [6, 7]. This has led to a limit of up to 42 days of storage in additive solutions in the US and Canada as well as in many European countries. These regulations do not consider the numerous biochemical, structural, inflammatory, and physiologic changes that occur in RBC units during storage (the “RBC storage lesion”), which may be deleterious to vulnerable populations [3, 7–12].

### Clinical studies examining RBC storage age and outcomes

Preclinical studies have showed that transfusion with RBCs stored for > 7 days can have adverse effects on microcirculatory flow and O_2_ utilization [5, 13]. Numerous clinical studies in humans, however, have observed conflicting results when comparing “older, less fresh” RBCs with “younger, fresh” transfused products including eight randomized controlled trials (RCTs), 32 observational studies, and several meta-analyses in varied patient populations [14–20]. Observational studies in critically ill patients have mostly reported an independent association between increased RBC age and organ failure or increased mortality [1, 21–25]. However, these studies, due to the nature of their design are often confounded by indication bias, as well as other sources of significant bias. Heterogeneous distribution of relatively old and young RBCs and the correlation of transfusion volumes and frequency of transfusion, which may reflect higher severity of illness, with storage age hinder evaluation of the independent effect of storage age on outcomes [26, 27].

The impact of transfusing older RBC units on morbidity and mortality, specifically in critically ill populations, thus remains a concern [22, 27–30].

Several randomized trials have been carried out in critically ill adults [31–35] and two trials have also been published in unique pediatric populations that have addressed the question [36, 37]; except for the ARIPI trial, all these were published after starting ABC PICU. While these trials provide evidence to guide RBC transfusion practices and blood banking policies in these populations, they did not find difference by RBC age with respect to the outcomes they were studying and do not provide the evidence required to guide practice in critically ill children – one of the only populations where fresh blood is being routinely being used [38]. The “Age of Blood in Children in Pediatric Intensive Care Unit” (ABC PICU) trial is a definitive study designed to address the question of whether transfusion of RBC units stored for 7 days or less reduces organ failure or death in critically ill children.

## Methods/design

### Study design

The ABC PICU trial is a large multicenter, international, double-blind, superiority, two-arm RCT. It will compare the risk of New or Progressive Multiple Organ Dysfunction Syndrome (NPMODS) between patients transfused RBCs of decreased storage age (length of storage ≤7 days) and those transfused standard-issue RBCs (stored for 2–42 days; expected average length of storage of about 17–21 days). The sample size of the trial is 1538 children and we are enrolling from a wide variety of pediatric hospitals in the US, Canada, France, Italy, and Israel. A summary of the protocol is provided in Table 1 (World Health Organization trial registration dataset) and in Additional file 1 (Standard Protocol Items: Recommendations for Interventional Trials (SPIRIT) Checklist).

**Table 1:** World Health Organization trial registration dataset

Primary registry and trial identifying number	ClinicalTrials.gov, ID: NCT01977547
Date of registration in primary registry	5 November 2013
Secondary identifying numbers	None
Source(s) of monetary or material support	1. National Heart, Lung and Blood Institute (Grant #1U01HL116383–01);2. Canadian Institutes of Health Research (Grant #126113), Ottawa, ON, Canada; 3. Comité National de la Recherche Clinique, Département de la Recherche Clinique et du Développement (DRCD), Assistance Publique – Hôpitaux de Paris, Ministère des Solidarités, de la Santé et de la Famille, France; 4. Ministère des Affaires Sociales et de la Santé, Paris, France (PHRC 14–0390); 5. The Ministère de la Santé et des Services Sociaux de la Province de Québec; and 6. Department of Pediatrics, Washington University in St. Louis, St. Louis, MO, USA
Primary sponsor	Investigator-initiated study Philip C. Spinella MD, FCCM Associate Professor of Pediatrics Division of Pediatric Critical Care Washington University in St. Louis St. Louis Children’s Hospital Campus Box 8116 One Children’s Place/NWT 10th fl. St. Louis, MO 63110, USA Phone: (314) 286–0858 Email: spinella_p@kids.wustl.edu
	Marisa Tucci MD Full Professor of Pediatrics Division of Pediatric Critical Care CHU Sainte-Justine Université de Montréal 3175 Côte Sainte-Catherine Montreal, QC Canada H3T 1C5 Phone: (514) 345–4931 × 3261 Email: marisa.tucci@recherche-ste-justine.qc.ca
Secondary sponsor(s)	Washington University in St. Louis St. Louis Children’s Hospital CHU Sainte-Justine Université de Montréal
Contact for public queries	Philip C. Spinella MD, FCCM, Washington University in St. Louis, St. Louis Children’s Hospital, Campus Box 8116, One Children’s Place/NWT 10th fl., St. Louis, MO 63110, USA Phone: (314) 286–0858 Email: spinella_p@kids.wustl.edu
	Marisa Tucci, MD, Sainte-Justine Hospital, 3175 Côte Sainte-Catherine, Montreal, QC, Canada H3T 1C5 Phone: (514) 345–4931 × 3261 Email: marisa.tucci@recherche-ste-justine.qc.ca
Contact for scientific queries	Philip C. Spinella MD, FCCM, Washington University in St. Louis, St. Louis Children’s Hospital, Campus Box 8116, One Children’s Place/NWT 10th fl., St. Louis, MO 63110, USA Phone: (314) 286–0858 Email: spinella_p@kids.wustl.edu
	Marisa Tucci, MD, Sainte-Justine Hospital, 3175 Côte Sainte-Catherine, Montreal, QC, Canada H3T 1C5 Phone: (514) 345–4931 × 3261 Email: marisa.tucci@recherche-ste-justine.qc.ca
Public title	Age of Blood in Children in Pediatric Intensive Care Units
Scientific title	The Age of Blood in Pediatric Intensive Care Units (ABC PICU) Randomized Clinical Trial
Countries of recruitment	Canada, US, France, Italy, Israel
Health condition(s) or problem(s) studied	Impact of red blood cell storage time on multiple organ dysfunction syndrome in critically ill children
Intervention(s)	Transfusion with either RBCs stored for ≤ 7 days or standard-issue red blood cells (oldest in inventory)
Key inclusion and exclusion criteria	Eligible for study: 1. a first RBC transfusion is requested within the first 7 days (168 h) of PICU admission; or 2. patient assessed pre-operatively and for whom PICU admission is planned post-operatively, and who is determined to definitively require a first RBC transfusion during surgery Inclusion criteria: critically ill pediatric patients who have an expected length of stay after transfusion in the ICU > 24 h based on the best judgment of the attending ICU staff Exclusion criteria: age at ICU entry < 3 days from birth or > 16 years of age; post-conception age < 36 weeks on admission to ICU; documented RBC transfusion within the 28 days prior to fulfilling the eligibility criteria; previously randomized in this study; weight < 3.0 kg on ICU admission; pregnant; conscious objection or unwillingness to receive blood products; not expected to survive beyond 24 h, brain death or suspected brain death; limitation or withdrawal of care decisions have been made; enrollment in another randomized clinical trial which has not been approved for co-enrollment; patients for whom autologous and/or directed donation RBCs will be provided; patients for whom the treating physician routinely and systematically requests RBC ≤ 14 days of storage; patients for whom there systematically exist RBC aliquoting policies that mandate the initial use of units stored for ≤ 14 days; on ECMO or plan to be immediately placed on ECMO at time of enrollment; patient predicted or presumed to require a massive transfusion (> 40 ml/kg of all blood components in a 24-h period) according to treating physician judgment; refusal by physician; inability to obtain consent; blood bank personnel experiences difficulties in securing blood products (difficult cross matches, rare blood groups, and diseases like IgA deficiency); insufficient number of ABO type compatible RBC units available in the blood bank at randomization with a storage time ≤ 7 days (minimum 1 unit regardless of patient age); all RBC units available for the patient are not leukocyte-reduced prior to storage
Study type	Multicenter, double blind, randomized controlled trial
Date of first enrollment	1 February 2013
Target sample size	1538
Recruitment status	Recruiting
Primary outcome	New or progressive multiple organ dysfunction syndrome
Key secondary outcomes	PICU and hospital mortality, 28-day, and 90-day all-cause mortality, nosocomial infections, PELOD-2 score, severe sepsis, septic shock, acute respiratory distress syndrome (ARDS), mechanical ventilation and PICU-free days
Ethics review	Approval obtained from the Institutional Review Board / Research Ethics Board of all participating sites and were in accordance with the institutional policies of the US Department of Health and Human Services in the US, provincial legislation in Canada, and appropriate entities in France, Italy, and Israel
Estimated completion date	June 2018

*ECMO* extracorporeal membrane oxygenation, *ICU* intensive care unit, *PELOD-2* pediatric logistic organ dysfunction version 2, *PICU* pediatric intensive care unit, *RBC* red blood cell

### Trial hypothesis

The hypothesis is that transfusion of RBC units stored for ≤ 7 days (definition of short storage) in critically ill children will reduce the proportion of patients who develop NPMODS, which includes death, within 24 days of randomization. We expect a reduction of at least 6% (33% relative risk reduction), from 18% in children receiving standard-issue RBCs to 12% in the “short-storage” group.

### Study population

Site eligibility requires validation that the site has the ability to perform the trial, confirmation by site survey that its blood bank(s) can provide short-storage RBC units as required as well as that the standard-issue RBCs will have a median storage age of at least 15 days. The ABC PICU study imposes minimal restrictions on patient eligibility, no controls on clinical practice and has opted to assess clinically important outcomes for pediatric critical illness.

#### Screening

**Fig. 1:**
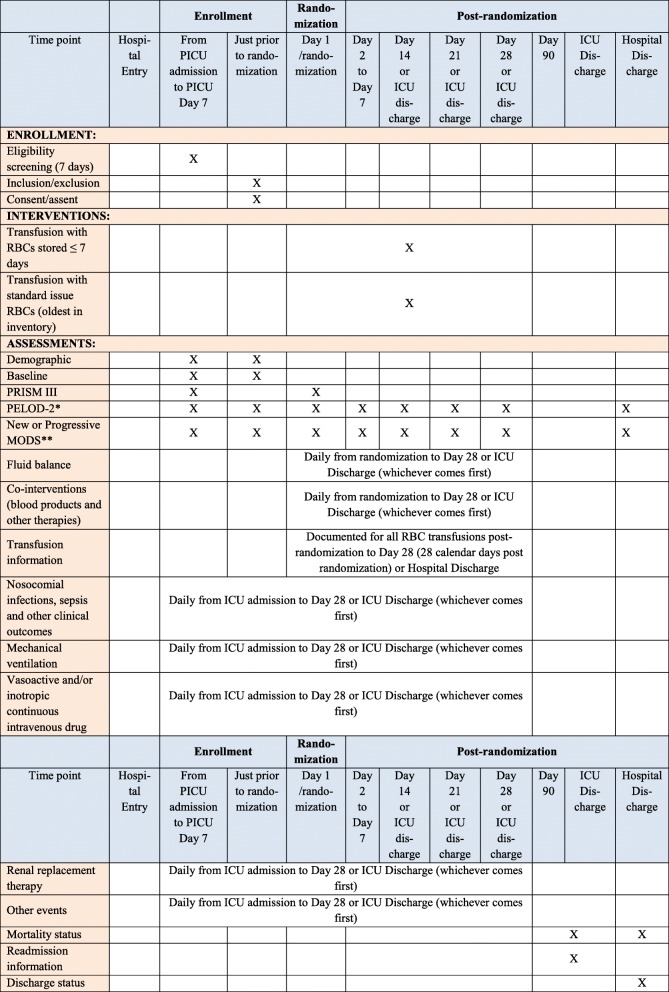
Screening, eligibility, consent, and randomization scenarios

Patients from 52 centers are screened and consented for randomization via three primary means (Fig. 1):

1. The clinical status and laboratory hemoglobin levels of PICU patients in ICU at high risk for RBC transfusion are monitored by research staff who verify eligibility, inclusion and exclusion criteria. If the patient meets all criteria, consent is obtained. Then, if RBC transfusion is ordered in the PICU (independent of the trial) within the first 7 days after admission, the patient is randomized. This period of eligibility is justified because the rate of NPMODS is low after 7 days in PICU (< 2%) [39]
2. A RBC transfusion is ordered in the PICU in a patient not identified via 1. Research staff verify inclusion and exclusion criteria. If the patient meets all criteria, consent is obtained and the patient is randomized
3. A patient who will require PICU admission post-operatively and for whom the surgeon deems a RBC transfusion will definitively be required intra-operatively. Research staff verify eligibility, inclusion and exclusion criteria. If the patient meets all criteria, consent is obtained pre-operatively. The patient is randomized when RBCs are requested for the operating room in preparation for surgery

Written informed consent from the patient or their legal guardian is required prior to randomizing a patient. Assent is obtained from the child whenever possible according to Institutional Review Board (IRB) requirements at each site.

#### Patient eligibility

A patient is considered eligible to participate in the trial if one of the following occurs: (1) a first RBC transfusion is requested within the first 7 days (168 h) of PICU admission; (2) a patient is assessed pre-operatively and, if PICU admission is planned, post-operatively, and determined to definitively require a first RBC transfusion during surgery. In either case, the patient must have an expected length of stay after transfusion in the PICU > 24 h based on the best judgment of the attending staff. Patients who meet any of the criteria listed in Table 2 are excluded.

**Table 2:** Exclusion criteria

1	Age at ICU entry < 3 days from birth or > 16 years of age
2	Post-conception age < 36 weeks on admission to ICU
3	Documented RBC transfusion within the 28 days prior to fulfilling the eligibility criteria
4	Previously randomized in this study
5	Weight < 3.0 kg on ICU admission
6	Pregnant
7	Conscious objection or unwillingness to receive blood products
8	Not expected to survive beyond 24 h, brain death or suspected brain death
9	Limitation or withdrawal of care decisions have been made
10	Enrollment in another randomized clinical trial which has not been approved for co-enrollment
11	Patients for whom autologous and/or directed donation RBCs will be provided
12	Patients for whom the treating physician routinely and systematically requests RBC ≤ 14 days of storage
13	Patients for whom there systematically exist RBC aliquoting policies that mandate the initial use of units stored for ≤14 days (ex: Pedi-Pack)
14	On ECMO or plan to be immediately placed on ECMO at time of enrollment
15	Patient predicted or presumed to require a massive transfusion (> 40 ml/kg of all blood components in a 24-h period) according to treating physician judgment
16	Refusal by physician
17	Inability to obtain consent
18	Blood bank personnel experiences difficulties in securing blood products (difficult cross matches, rare blood groups, and diseases like IgA deficiency)
19	Insufficient number of ABO type compatible RBC units available in the blood bank at randomization with a storage time ≤ 7 days (minimum 1 unit regardless of patient age)
20	All RBC units available for the patient are not leukocyte-reduced prior to storage

Exclusion criteria # 1 to 17 are ascertained by the research staff with the assistance of the attending ICU team

Exclusion criteria # 18 to 20 are ascertained by blood bank personnel

*ECMO* extracorporeal membrane oxygenation, *ICU* intensive care unit, *RBC* red blood cell

#### Clinical and outcome information

**Fig. 2:**
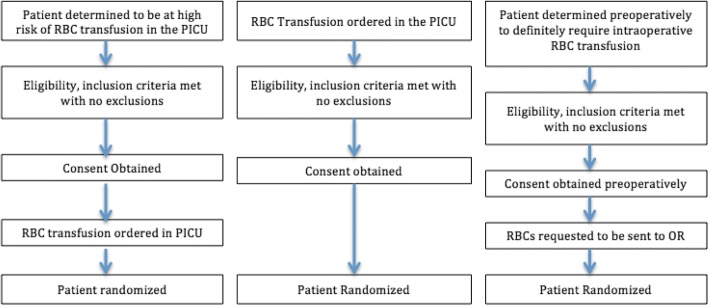
Standard Protocol Items: Recommendations for Interventional Trials (SPIRIT) Figure: schedule of enrollment, interventions, and assessments

A schedule of enrollment, interventions and assessments is reported in Fig. 2. ICU data collected is listed in Table 3. Baseline data at admission includes co-morbidities, type of ICU admission, blood type, and hemoglobin prior to first RBC transfusion. Clinical and outcome data, as well as the Pediatric Logistic Organ Dysfunction version 2 (PELOD-2) score and specific MODS information, are collected daily through day 7 after randomization, then on days 14, 21, and 28. This data elements are also collected at ICU discharge if the patient is discharged prior to day 28. Proposed duration of monitoring of RBC transfusion (study intervention), co-interventions, and follow-up for the primary outcome NPMODS is 28 days following randomization or until PICU discharge or death, whichever happens first.

**Table 3:** ICU data collected

• Demographic data	
• Baseline data	
• PRISM III score	
• PELOD-2 score	
• Clinical data for organ dysfunctions	
• Red blood cell transfusion information	
• Mechanical ventilation	
• Evidence of infection	
◦ nosocomial pneumonia	
◦ blood stream infection	
◦ sepsis	
◦ severe sepsis	
◦ septic shock	
• Acute respiratory distress syndrome	
• Deep venous thrombosis	
• Any transfusion reaction	
• Critical care interventions	
◦ length of mechanical ventilation	
◦ hemodynamic support	
◦ renal replacement therapy	
◦ fluid balance per day	
◦ proportion of patients receiving erythropoietin	
◦ vasoactive drugs	
◦ mild to moderate hypothermia treatment	
◦ systemic corticosteroids	
◦ insulin administration	
◦ starch colloids and/or gelatins	
◦ plasmapheresis	
◦ Molecular Adsorbent Re-Circulating System (MARS)	
• Tranfusion interventions other than RBC administration (use and volume per kg)	
◦ frozen plasma	
◦ platelets	
◦ cryoprecipitate	
• Discharge status	
• Mortality status	
• Readmission information	

*ICU* intensive care unit, *PELOD-2* Pediatric Logistic Organ Dysfunction version 2, *PRISM III* Pediatric risk of mortality

### Interventions

The trial interventions are either short-storage RBCs (stored for ≤ 7 days) or standard-issue RBCs. All RBC units are prepared in accordance with international standards. All blood products including the RBCs studied in this trial are supplied by the hospital blood bank according to applicable local regulations. Only pre-storage leukoreduced RBC units are used in this trial as per local standards of care.

### Randomization and treatment allocation

Patients are randomized to receive either (1) short-storage RBCs (stored for ≤ 7 days) or (2) standard-issue RBCs (stored for 2 to 42 days). Inventory management requires that randomization can occur only when there are RBCs ≤ 7 days *and* RBCs ≥ 5 days available to meet the crossmatch request.

The randomization process is being done using an Internet-based system and consists of an Internet-based, computer-generated random listing of treatment allocation using a pre-established algorithm. Allocation is in a 1:1 ratio. Patients are stratified at randomization according to center and age (≤ 28 days after the day of birth, 29 to 365 days, and > 1 year). Stratification by site and age is employed as unbalanced treatment allocation is possible given the diversity in case mix within each of the participating PICUs. In order to conceal future allocation, three sizes of block permutation (2, 4, and 6 patients per block) are randomly used in each stratification tree via a computer-generated randomization scheme.

### Blinding

Blinding is used not only in the allocation process (concealment of randomization), but also in the intervention allocation (fresh versus standard delivery). The blood bank technologist verifies the expiry date of the RBC unit prior to its release, registers the date of collection of the RBC unit delivered and refrains from releasing information on the storage times of RBC units to clinical personnel. Thus, physicians, nurses, other caregivers and research staff are not given any information regarding individual entries from the computer-generated random list.

To blind clinicians and research personnel, opaque stickers are placed on expiration dates on the labels affixed to bags of RBC units in the blood bank before any RBC unit is delivered to a patient participating in ABC PICU. In France, blinding occurs by re-printing a blood-bag label on which the expiry/collection date was no longer showing. Accidental un-blinding of the RBC unit expiration date is documented and reported via the protocol deviation process. The data of study patients with accidental un-blinding will be included in the intent-to-treat (ITT) analysis.

Outcome evaluation, diagnosis of NPMODS and the determination of all scores are done by research assistants who are unaware of treatment allocation.

### Compliance

#### Measures to ensure adherence

Measures instituted to highlight trial participation and to maximize protocol adherence include: (1) provision of institute-specific protocols for inventory management of RBC units for the trial and tracking of patients enrolled in the trial, (2) monitoring of RBC supply in some sites to ensure adequate supplies are available for randomization, (3) audits on the age of RBCs entered in the system to identify sites that may have issues to address, and (4) mechanisms to allow for appropriate RBCs (short storage versus standard issue) to be transferred from other local sites or from blood providers. Patients who receive no transfusion in the 28 days after randomization will be excluded from the per-protocol analysis. It might occur very rarely that a patient in the standard-issue arm is moved to the short-storage arm. If this was to occur, these patients will also be considered as non-compliant and will be removed from the per-protocol primary analysis but will be kept in the ITT analysis.

#### Compliance with the intervention

Patients in the short-storage arm of the study are considered adherent to protocol if 80% or more of transfused RBCs are stored for ≤ 7 days and if they receive no RBC unit stored for > 14 days during the 28-day follow-up period. If not, data for that subject will be removed from the per-protocol analysis.

The clinical team can administer any available RBCs regardless of storage time to patients who become unstable and have transfusion requirements that do not allow for adherence to the protocol.

The decision to withhold or to withdraw critical care will not be considered an exclusion criterion if it occurs after a patient enters the trial. These cases will be kept in the ITT analysis.

### Outcomes

#### Primary outcome measure

The primary outcome measure is rate of 28-day NPMODS, defined as the proportion of patients who die during the 28 days after randomization or who develop NPMODS including mortality. For patients with no organ dysfunction at randomization, “New MODS” is the development of two or more concurrent organ dysfunctions; for patients with one organ dysfunction at randomization; New MODS is the development of at least one other concurrent organ dysfunction; patients with MODS (i.e., concurrent dysfunction of two or more organ systems) at randomization can develop “Progressive MODS” defined as the development of at least one additional concurrent organ dysfunction. All deaths are considered Progressive MODS. NPMODS is monitored daily for the first 7 days following randomization and then weekly up to 28 days or PICU discharge because it is almost never observed beyond this time in children [39].

#### Secondary outcome measures

Secondary outcomes include PICU and hospital mortality, 28-day, and 90-day all-cause mortality. Nosocomial infections will be recorded, including nosocomial pneumonia and bloodstream infection. Other secondary outcomes include PELOD-2 score, which measures the severity of MODS [40, 41], severe sepsis, septic shock, acute respiratory distress syndrome, mechanical ventilation and PICU-free days.

#### Adverse events and serious adverse events

The list and definitions of adverse events and transfusion reactions as well as reporting timelines are described in Additional file 2.

### Sample size

Patient eligibility criteria and short-storage definition (7-day cutoff) in ABC PICU will be similar to those used in preliminary studies (Table 4) [1, 23]. Based on this prior work and on a large survey of North American intensivists [38], the incidence of NPMODS is expected to be 18% in the control group and 12% in the short-storage group and the relative risk is expected to be 33%. Sample size calculations based on these estimates for two independent proportions (chi-square) using a two-tailed *α* of 0.05 and a (1 − *β*) of 0.90 yield an estimate of 769 patients per arm (total: 1538) [42]. The ABC PICU Trial Steering Committee, the CCCTG (www.ccctg.ca), and the PALISI Network (www.palisi.org) support these estimates and the choice of a 33% relative risk difference because it is considered clinically important and sufficiently significant to change practice. The proportion of patients lost to follow-up is expected at 1.7% based on results of the TRIPICU study. The sample size for the ABC PICU trial is, therefore, 769 patients per arm (total: 1538) [42].

**Table 4:** Estimates for the absolute risk reduction expected in the ABC PICU trial

	Analytic cohort study: Gauvin et al. [1]	Descriptive cohort study: Karam et al. [2]
Storage time cutoff for “fresh” RBC unit	7 days	7 days
PICU expected length of stay	> 24 h	> 48 h
Hemodynamically unstable patients	No	Yes
NPMODS in transfused patients	15%	39.2%
Odds ratio for development of NPMODS in older versus fresher (confidence interval)	1.39 (0.42–4.61)	1.54 (0.80–2.96)
Estimated risk in experimental group 1/*OR* = (*p*1/(1 − *p*1))/(*p*0/(1 − *p*0))	11%	22%

*MODS* multiple organ dysfunction syndrome, *NPMODS* new or progressive multiple organ dysfunction syndrome, *OR* odds ratio, *PICU* pediatric intensive care unit, *RBC* red blood cell, *RCT* randomized controlled trial

### Statistical analyses

#### Baseline characteristics

Baseline characteristics of patients, intervention, and co-interventions in both study arms will be assessed using frequency distributions and univariate descriptive statistics including measures of central tendency and dispersion. Mean (± standard deviation) and median (interquartile range) will be used to report data as appropriate. Percentages will be reported for categorical data. Any known clinical risk factor, whether or not there is a statistically significant imbalance, will be considered for adjusted analyses of primary and secondary outcomes.

#### Intervention and co-interventions

Post-randomization characteristics of the intervention (short storage versus standard issue RBC units) and major co-interventions (platelets, plasma, fluid balance, etc.) will be presented using frequency distributions with measures of central tendency and dispersion, and analyzed using relative risks and 95% confidence interval (CI) for dichotomous data (e.g., proportion transfused with platelets) and Wilcoxon Rank Sum tests for difference in continuous data (e.g., difference in median platelet use).

#### Analysis of the primary outcome measure

**Fig. 3:**
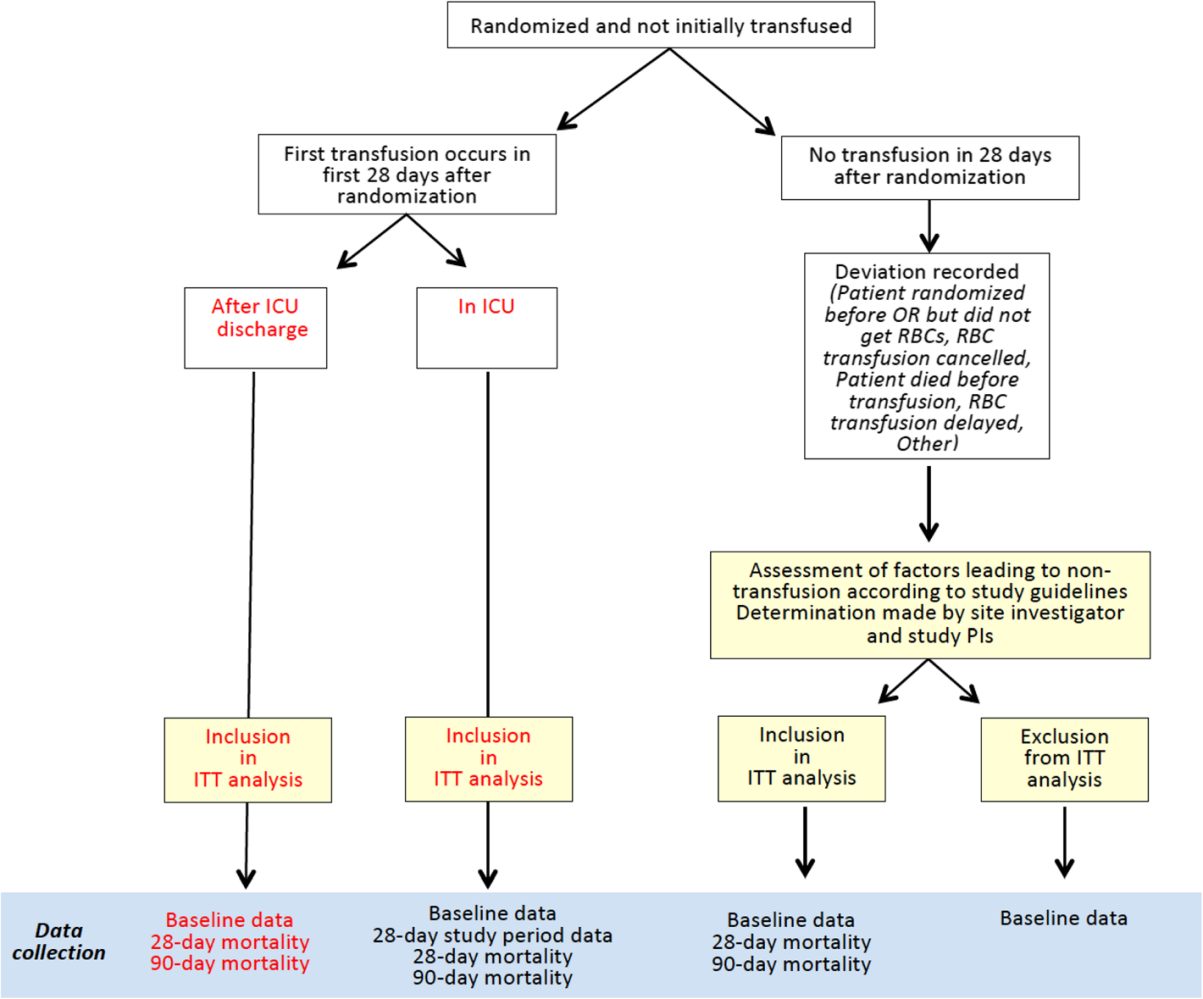
Intent-to-treat (ITT) analysis

As this is an effectiveness trial, the analysis of the primary outcome measure will be conducted on an ITT approach. Data from all participants enrolled will be analyzed according to the intervention to which they were allocated, regardless of whether it was received or not (Fig. 3). The primary outcome (i.e., the effect of treatment, short storage versus standard issue, on development of NPMODS), will be analyzed using an unadjusted chi-square. The principal effect measure will be an unadjusted relative risk reduction with a 95% CI. A per-protocol analysis of the primary outcome measure will also be done. Sensitivity analysis will be performed that excludes patients in the short-storage group who receive RBCs stored for > 7 days and patients in the standard-issue arm who receive RBCs stored for ≤ 7 days. Hypothesis testing for the primary analysis will be carried out with an overall level of significance set using a *p* value < 0.05, taking into account one interim analysis with the *p* value being determined by the O’Brien-Fleming stopping rule [43]. All *p* values will be reported as two-sided.

Secondary analyses of the primary outcome (NPMODS) include a logistic regression model to further elucidate the measure of effect while adjusting for known prognostic factors or those thought to be associated with multiple organ failure. For associated prognostic risk factors, independent covariates, such as center, age, gender, and co-morbid illnesses, and severity of illness scores, will be added to all logistic models. Potential confounding factors, if clinically relevant, will be considered for inclusion into logistic models. Continuous risk factors (e.g., pediatric risk of mortality (PRISM) III, number of transfusions per patient) will be entered into the models as a continuous measure rather than categorical to improve statistical efficiency. Regression diagnostics will be performed on all models. We will plot continuous variables and check for linearity before including in regression models. Variables not meeting a reasonable linearity assumption will be transformed. Odds ratios will be estimated from coefficients and CIs will be constructed using Robins-Greenland procedures [44]. Kaplan-Meier curves will be compared using a log-rank test followed by proportional hazards modeling for NPMODS rates: this analysis will compare the length of time between randomization and onset of NPMODS.

#### Analyses of secondary outcome measures

As with the primary outcome, secondary outcome measures will be analyzed by an ITT approach. The effect of treatment on dichotomous secondary outcomes will be determined by calculating relative risk reduction and relative risks followed by logistic regression procedures. Continuous outcome measures, such as the PELOD-2 score, mechanical ventilation days, ventilator-free days, PICU length of stay, and PICU-free days, will be analyzed using either parametric procedures (independent *t* test) or non-parametric procedures (Wilcoxon Rank Sum). The influence of treatment groups (short storage versus standard issue) on categorical variables, including mortality and infectious complications, will be analyzed using either parametric or non-parametric procedures followed by multiple comparison procedures (e.g., sequentially rejective Bonferroni procedure), as deemed appropriate. Interactions will be sought between RBC storage time and other parameters with respect to the primary outcome such as the severity of organ dysfunction, the highest number of organ dysfunctions, and the PELOD-2 score (patients who die will be assigned the worst possible NPMODS score at the time of death). This includes an analysis based on the number of units transfused. Per-protocol analyses undertaken for secondary outcomes will be only exploratory.

#### Subgroup analyses

Subgroup analyses are planned for: (1) illness category (cardiac surgery, general surgery, trauma, medical), (2) volume/kg of RBCs transfused (analyzed by quartiles and other methods), (3) severity of illness at baseline, as evaluated by the PRISM III score [45], (4) stable versus unstable patients at the time of first transfusion (as defined in the TRIPICU study) [46], and (5) ABO type.

The analytic approach used for all subgroup analyses will be ITT. Interactions between treatment group in subgroup categories specified above will be calculated. Interactions will be assessed by adding the treatment, subgroup of interest (categorized), and its interaction term (treatment × subgroup) in a multivariate logistic regression model. We recognize the limitations of subgroup analyses (low power, type I error, difficulties in interpretation) [47]. These analyses will be hypothesis-generating and hypothesis-supporting in nature.

#### Excluded patients

A limited analysis will be conducted on all patients meeting the inclusion and exclusion criteria but who were not randomized, using data in the screening log, to ascertain if these patients are different from those randomized in the ABC PICU trial.

### Trial management

#### Data management

Data management is performed at the Ottawa Health Research Institute under the supervision of the study statistician (DF) at this site. Data is entered on site in the web-based electronic case report form (eCRF). During the validation phase, the case report form (CRF) and entries were considered adequate if the frequency of discordance was lower than 2% in the CRF. The Data Management Center (DMC) and the Coordinating Centers (Sainte-Justine Hospital, University of Montreal, and St. Louis Children’s Hospital, Washington University School of Medicine) are responsible for data quality assurance done through eCRF (via regular data extraction) and queries. A second satellite Data Management Center (DMC) was created in St. Louis that involved a study statistician (KS). The St. Louis DMC has three overarching responsibilities: (1) development and implementation of an automated query generation system, (2) Data and Safety Monitoring Board (DSMB) report completion, and (3) adverse event monitoring and reporting. Data is uploaded every other week for the duration of the study at this site; all data is automatically encrypted when the transfer takes place.

#### Interim analysis

The DSMB of the ABC PICU trial requested that an interim analysis be done when enrollment and completed data collection reached 50% of recruitment targeted. The interim statistical analysis will compare NPMODS rates in the short-storage and standard-issue groups, using O’Brien-Fleming stopping rules [43], with a two-tailed *p* value. Stopping rules are based upon safety concerns as assessed by the DSMB. The DSMB of the ABC PICU trial could consider terminating enrollment if the statistical analysis showed a statistically significant difference. Both positive and negative findings from the ABC PICU trial are considered of clinical interest.

#### Study monitoring

The DSMB for ABC PICU was established by the National Heart Lung and Blood Institute (NHLBI) to monitor data and oversee patient safety in this study and convenes twice a year. The principal investigators, staff from the DMC and NHLBI participate in the meetings as non-voting members. Study monitoring methods vary according to country. For all participating centers throughout the US, Canada, and Europe, 100% of eCRFs are audited through automated query process using pre-determined ranges flagging suspicious or out-of-range values.

For US participating centers, as well as for Italy and Israel, there are a total of three scheduled site visits (with the exception for those sites that on-boarded in 2017 where there may only be one or two visits due to time restraints). These visits are undertaken following the enrollment of, and completion of, 28-day data of the first two randomizations at the study interim, and a visit is planned at study close-out. Additional site visits are carried out at any time as deemed necessary. For international sites, a site initiation visit occurred prior to on-boarding to review the US regulations, study design and eCRF for clarity. During monitoring visits, a review of 100% of regulatory documents, IRB/REB paperwork, consent, and eligibility for compliance are done. A review of all data queries and corrections received to date is completed to ensure resolution to the extent possible. At each visit 20–30% of charts are reviewed for primary and secondary outcomes. In Canada, site initiation was conducted by teleconference prior to screening start and included members of the ICU research team, as well as blood bank personnel. The Canadian Coordinating Center audited data electronically by extracting and questioning sites on out-of-range values, as well free-text answers The Coordinating Center also verified coherence and logic between variables. The Coordinating Center did not exclude the option of conducting for-cause monitoring visits; however, none have been required so far. In France, the CHU de Lille conducted on-site initiation visits prior to start of screening.

#### Close-out and access to data

All data and source documentation will be stored in a secure storage facility for 7 years from the time of study close-out. Datasets must be submitted to the study NHLBI study program official no later than 3 years after the end of the clinical activity (final patient follow-up, etc.) or 2 years after the main paper of the trial has been published, whichever comes first. Data is prepared by the study coordinating center and sent for review prior to release. The NIH and NHLBI expect the timely release and sharing of data to be no later than the acceptance for publication of the main findings from the final dataset.

## Discussion

RBCs are the most frequently transfused blood product with approximately 85 million RBC units transfused worldwide per year, of which 12–16 million units were transfused in the US [48]. The blood banking system is organized to issue the oldest RBC units first to minimize wastage of this valuable and limited resource. Thus, RBCs are transfused in critically ill children even if they have been stored for up to 42 days, despite laboratory evidence suggesting significant changes to cell structure and no strong evidence that they remain effective and safe. There is a widely held belief that the freshest RBCs possible could benefit critically ill children. Indeed, for certain patients, such as children undergoing cardiac surgery, there are already agreements with blood banks to use only fresh RBCs even though there is no evidence to support such practice [38]. Further, this practice raises ethical concerns regarding inequitable use of available RBC units.

Several recent RCTs in adults, including the Red Cell Storage Duration Study (RECESS) trial, the Age of Blood Evaluation (ABLE) randomized controlled trial, the Informing Fresh versus Old Red Cell Management (INFORM) trial, and the Standard Issue Transfusion versus Fresher Red-Cell Use in Intensive Care (TRANSFUSE) trial, have compared RBC units aged less than 10 days with transfusion of 14- to 42-day-old RBC units in various critically ill populations [31–34]. In these studies, as well as in a recent systematic review [35], there was no survival advantage in transfusing “fresher” blood. However, findings from adult studies may not be applicable to children because host characteristics and developmental differences may have a significant impact on the risks and benefits of anemia and transfusion in this population [49].

Two RCTs have been published in pediatric populations. The Tissue Oxygenation by Transfusion in Severe Anemia With Lactic Acidosis (TOTAL) trial failed to show a difference in elevated blood lactate levels among children with severe anemia who received RBCs with a more prolonged storage time [50]. Most of the children in this trial had malaria or sickle cell disease. Generalizability of the results of this trial is questionable because RBC transfusion practices, case mix, and the etiology of shock are different in more developed countries. The Age of Red Blood Cells in Premature Infants (ARIPI) trial investigated the effects of RBC storage in premature neonates and demonstrated that fresher RBCs did not improve a composite outcome measure that included major neonatal morbidities, including necrotizing enterocolitis, retinopathy of prematurity, bronchopulmonary dysplasia, and intraventricular hemorrhage, as well as death [37]. Except for death, these outcomes are never seen in non-premature PICU patients.

The ABC PICU trial is designed to definitively address this question in a large general PICU population representative of critically ill children. It will either support the wide adoption of optimal RBC transfusion practices in critically ill children. The primary outcome is clinically relevant and widely accepted. There is consensus among members of the Pediatric Acute Lung Injury (PALISI) and the Canadian Critical Care Trials Group Networks that results would be clinically meaningful and practice change would be justified if ABC PICU shows superiority for fresher RBC units. Clinically, important secondary outcomes will be evaluated and pertinent subgroup analyses are planned. In addition, some exploratory analyses are being considered that will involve examining the main effect (storage duration) as a function of RBC dose. Given that multiple transfusions with RBCs of different ages will occur in both treatment arms and because 7 days is an arbitrary cutoff point, analyses are planned that will assess whether cut-points other than 7 days are preferable.

### Trial status

The ABC PICU trial is evaluating whether fresher RBCs can reduce NPMODS in a large international cohort of critically ill children. Patient recruitment began in February 2013 and is currently ongoing (protocol version: 5 May 2016). Recruitment started at the two coordinating centers in Canada and the US as well as in four vanguard sites in the US. There are 30 sites in the US, 10 sites in Canada, eight sites in France, three sites in Italy and one site in Israel. Recruitment is expected to continue until June 2018. Results from this trial should influence transfusion practice regardless of the outcomes. If no difference is found, this will reassure clinicians and transfusion medicine specialists regarding the safety of the current system of issuing the oldest RBCs in the inventory and will discourage clinicians from preferentially requesting RBCs of decreased storage age in critically ill children. If a difference is found, this will indicate that fresher RBCs improve outcomes in the PICU population and would justify the use of fresher RBC use in critically ill children.

## Additional files

Additional file 1: Standard Protocol Items: Recommendations for Interventional Trials (SPIRIT) Checklist. (DOC 121 kb)

Additional file 2: Definitions, severity grading, and reporting of adverse events. (DOCX 97 kb)

## Abbreviations

ABC PICU: Age of blood in children in PICU
CI: Confidence interval
CRF: Case report form
DMC: Data Management Center
DSMB: Data and Safety Monitoring Board
eCRF: Electronic case report form
ICU: Intensive care unit
IRB: Institutional Review Board
MODS: Multiple organ dysfunction syndrome
NHLBI: National Heart Lung and Blood Institute
NPMODS: New or Progressive Multiple Organ Dysfunction Syndrome
PELOD-2: Pediatric Logistic Organ Dysfunction version 2
PICU: Pediatric intensive care unit
PRISM: Pediatric risk of mortality
RBC: Red blood cell
RCT: Randomized controlled trial
REB: Research Ethics Board
