# Supplementary material for: The age of blood in pediatric intensive care units (ABC PICU): study protocol for a randomized controlled trial
Source: Trials. 2018 Jul 28;19:404. doi: 10.1186/s13063-018-2809-y (PMC6064163; doi:10.1186/s13063-018-2809-y)
Supplement: Supplementary file 2 — Definitions, severity grading, and reporting of adverse events. (DOCX 97 kb) [file 13063_2018_2809_MOESM2_ESM.docx]

**Additional file 2: Definitions, severity grading and reporting of adverse events**

Adverse event screening starts at randomization and stops after the patient reaches 28 days post randomization or 72 hours after the last study transfusion, whichever is later and provided the patient is still in hospital. Transfusion Associated Graft versus Host Disease screening is done throughout the hospitalization (even if greater than 28 calendar days post randomization).

All serious adverse events are evaluated for expectedness and/or relatedness. For expectedness, serious adverse events that are part of the natural history of the primary disease process or complications that are reasonably expected in association with critical illness or that are a consequence of the treatments required are reported as expected serious adverse events. If the occurrence of any serious adverse event is considered significantly unusual for a given patient's clinical course, it may be reported as unexpected; a medical monitor reviews this assessment and may contact the site investigator for further information. Relatedness is determined only with regard to participation in the trial and is not evaluated with regard to the RBC transfusion itself, since this is not the study intervention.

All grade 3-5 serious adverse events require documentation of resolution.

The Data Coordinating Center reports each adverse event and serious adverse event to the National Heart, Lung and Blood Institute who forwards them to the DSMB Chair according to the reporting timelines described in the figure below.


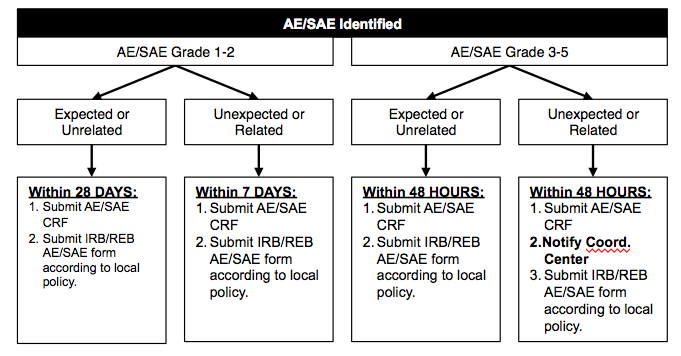


AE: adverse events; IRB: institutional review board; REB: research ethics board; SAE: serious adverse event

**Severity grading and definitions for serious adverse events**

An adverse event or suspected adverse reaction is considered serious if it results in a grade 4 or 5 as defined by the following: a) grade 4: an adverse event, that is life threatening or prolongs the existing hospitalization; b) grade 5: death.

| Adverse Event | 1 | 2 | 3 | 4 | 5 |
| --- | --- | --- | --- | --- | --- |
| **Acute Respiratory Distress Syndrome (ARDS)**  Definition:   1. PaO_2_/FiO_2_ ratio ≤ 300 mmHg or SpO2/FiO2 ratio ≤253 mmHg that is not explained by cardiac failure and fluid overload.   **AND**   1. Confirmation of bilateral opacities (perihilar infiltrates alone are not considered) on chest imaging report not explained by cardiac failure and fluid overload. |  |  | Prolongation of hospitalization directly attributable to the event and/or persistent or significant disability or incapacity | Life-threatening respiratory compromise.  Major intervention required following the transfusion (extracorporeal support e.g. ECMO) to prevent death | Death |
| **Deep vein thrombosis***  Definition :   1. Criteria for clinically suspected or proven deep vein thrombosis will include at least 1 of the following symptoms: superficial collateral circulation, jugular swelling, vena cava syndrome, edema, pain, inflammation of a limb, fever or signs of a thrombotic complication (e.g., pulmonary embolism), as described by Dubois et al. 2. Criteria to identify deep vein thrombosis as diagnosed by ultrasonography will include direct visualization of the thrombus, non-compressibility of the vessel, incomplete filling of the vein and appearance of respiratory variations on venous flow as described by Prandoni et al.   *Symptomatic and asymptomatic deep vein thromboses will both be reported | Venous thrombosis (e.g., superficial thrombosis)  No intervention indicated. | Venous thrombosis (e.g., uncomplicated deep vein thrombosis),  Anticoagulation indicated | Venous thrombosis,  Intervention indicated other than anticoagulation (lysis, invasive procedure) | Venous thrombosis associated with embolic event.  Life-threatening (e.g., pulmonary embolism, cerebrovascular event, arterial insufficiency); hemodynamic or neurologic instability; urgent intervention indicated | Death |
| **Transfusion Associated Graft vs. Host Disease**  Definition:  Due to the complexity of this diagnosis, report as present if medical record shows as diagnosed by clinical team within 28 days after the last transfusion. | Skin :Macular or papular eruption or erythema without associated symptoms  AND/OR  Minimal GI symptoms  AND/OR  Bilirubin >ULN – 1.5 x ULN | Skin :Macular or papular eruption or erythema covering <50% of body surface area (BSA)  AND/OR  Moderate GI symptoms  AND/OR  Bilirubin >1.5 – 3.0 x ULN | Skin :Severe, generalized erythroderma or macular, papular or vesicular eruption covering ≥50% BSA  AND/OR  Severe GI symptoms; inability to aliment  by GI tract requiring enteral parenteral nutrition);  AND/OR  Bilirubin >3.0 – 10.0 x ULN | Life-threatening consequences.  Skin :Generalized exfoliative, ulcerative, or bullous dermatitis  AND/OR  Very severe GI symptoms with significant diarrhea and bleeding.  AND/OR  Bilirubin >10.0 x ULN | Death |
| **Hemolytic transfusion reaction**  Definition  Requires presence of hemoglobinuria or hemoglobinemia, new or unexplained by underlying condition, observed from the beginning of the transfusion up to 4 hours after end of the transfusion.   - - Hemoglobinuria: macroscopic or microscopic hemoglobinuria, red, pink or very dark urine with positive test for hemoglobin in urine, observed from the beginning of the transfusion up to 4 hours after it was completed   - Hemoglobinemia: blood level of free hemoglobin above normal range or positive direct Coombs test (also know as the direct antiglobulin test or DAT)   - At least one of the following symptoms/signs.  1. Fever (> 38°C) de novo. 2. Dyspnea de novo. 3. Hypotension and/or tachycardia de novo. 4. Anxiety/agitation de novo. 5. Pain de novo | Laboratory evidence of hemolysis only (e.g., direct antiglobulin test; DAT; Coombs’; schistocytes; decreased haptoglobin) | Evidence of hemolysis and >=20 g/L (2g/dL): decrease in hemoglobin | Transfusion or medical intervention indicated (e.g., steroids) | Life-threatening consequences; urgent intervention indicated | Death |
| **Hyperkalemia**  Definition:  Blood level of potassium > 5.5 mmol/L.  ULN = Upper Limit Normal | >ULN – 5.5 mmol/L  And  Asymptomatic | >ULN – 5.5 mmol/L  And  ECG changes without hemodynamic instability | >ULN – 5.5 mmol/L  And  Hemodynamic instability | >ULN – 5.5 mmol/L  And  Life-threatening consequences | >ULN – 5.5 mmol/L  And  Death |
| **Hypocalcemia**  Definition:  An ionized calcium concentration < 0.8 mmol/L (<3.2 mg.dL) | Ionized calcium <0.8 mmol/L;  And  Asymptomatic | Ionized calcium <0.8 mmol/L;  And  ECG changes without hemodynamic instability | Ionized calcium <0.8 mmol/L;  And  Hemodynamic instability or tetany | Ionized calcium <0.8 mmol/L;  And  Life-threatening consequences | Ionized calcium <0.8 mmol/L;  And  Death |
| **Major allergic reaction**  Definition (including major anaphylactic reaction):  Disorder characterized by an adverse local or general response (Type 1 hypersensitivity reaction) from exposure to an allergen that includes at least 1 of the following (within 24 hours):   - Cardiac Arrest - Generalized allergic reaction or anaphylactic reaction de novo - Angio-edema (facial and/or laryngeal) de novo (as reported by nurses). - Upper airway obstruction de novo. - Dyspnea de novo, wheezing de novo. - Hypotension de novo, shock de novo. - Precordial pain or chest tightness de novo. - Cardiac arrhythmia de novo. - Loss of consciousness de novo. | Transient flushing or rash, drug fever <38 degrees C (< 100.4 degrees F), intervention not indicated | Intervention or infusion interruption indicated, responds promptly to symptomatic treatment (e.g., antihistamines, NSAAIDS, narcotics), prophylactic medications indicated for <= 24 hrs | Symptomatic bronchospasm, with or without urticaria; parenteral intervention indicated; allergy-related edema/angiedema; hypotension.  Prolonged (e.g. not rapidly responsive to symptomatic medication and/or brief interruption of infusion); recurrence of symptoms following initial improvement; hospitalization indicated for clinical sequelae (e.g., renal impairment, pulmonary infiltrates) | Life-threatening consequences; urgent intervention indicated | Death |
| **Nosocomial pneumonia**  Definition :  A patient with a new or progressive radiographic infiltrate, along with a high clinical suspicion of pneumonia plus a definite cause established, must be intubated for at least 48 hours and diagnosis is made after patient has received a transfusion.  Microbiologically confirmed: The patient must have:  A new or progressive radiographic infiltrate,  **along with**  A high clinical suspicion of pneumonia  **plus**  A definite cause established by   - Recovery of a probable etiologic agent from an uncontaminated specimen (blood, pleural fluid, transtracheal aspirate, or transthoracic aspirate); **or** - Recovery from respiratory secretions of a likely pathogen that does not colonize the upper airways (e.g., Mycobacterium tuberculosis, Legionella species, influenza virus, or Pneumocystis jiroveci (carinii); **or** - Recovery of a likely/possible respiratory pathogen in high concentrations using quantitative cultures of a lower respiratory tract sample (endotracheal aspirate, BAL, or protected specimen brush); **or** - Positive serology |  |  | Prolongation of hospitalization directly attributable to the event and/or persistent or significant disability or incapacity | Life-threatening respiratory compromise.  Major intervention required following the transfusion (extracorporeal support .e.g. ECMO) to prevent death | Death |
| **Transfusion-associated cardiac overload (TACO)**  Definition:  TACO is a caused de novo by a transfusion as a result of fluid overload (positive fluid balance > 20/mL/kg in the last day) that appears within 6 hours after the end of a transfusion, with at least one of the following criteria:   1. Dyspnea or cyanosis de novo (as reported by nurses). 2. Pulmonary edema de novo. 3. Tachycardia de novo. 4. Hypertension de novo. | Radiologic findings only; minimal dyspnea on exertion | Moderate dyspnea on exertion  Medical intervention (e.g. symptomatic treatment) required but lack of such would not  result in permanent damage or impairment of a body function. | Severe dyspnea or dyspnea at rest; oxygen indicated.  Inpatient hospitalization or prolongation of hospitalization directly attributable to the event and/or persistent or significant disability or incapacity  OR  A medical or surgical intervention is necessary to preclude permanent damage or impairment of a body function. | Life-threatening respiratory or hemodynamic compromise  Major intervention required following the transfusion (vasopressors, intubation, ventilator support) to prevent death | Death |
| **Transfusion-related acute lung injury TRALI**  Definition:  TRALI is caused de novo by a transfusion, and appearing during or less than 6 hours after the end of a transfusion.  New onset or worsening of pulmonary function with hypoxemia that satisfies the international criteria for ALI (PaO_2_/FiO_2_ <300 mm Hg)  **AND**  Chest x-ray consistent with pulmonary edema | Radiologic findings only; minimal dyspnea on exertion. | Moderate dyspnea on exertion  Medical intervention (e.g. symptomatic treatment) required but lack of such would not  result in permanent damage or impairment of a body function. | Severe dyspnea or dyspnea at rest; oxygen indicated  Prolongation of hospitalization directly attributable to the event and/or persistent or significant disability or incapacity  OR  A medical or surgical intervention is necessary to preclude permanent damage or impairment of a body function. | Life-threatening respiratory compromise.  Major intervention required following the transfusion (vasopressors, intubation, ventilator support) to prevent death | Death |

| Adverse Event | 1 | 2 | 3 | 4 | 5 |
| --- | --- | --- | --- | --- | --- |
| **Septic shock**  **Definition :**  Septic Shock is defined as sepsis + cardiovascular dysfunction.  Sepsis is defined as SIRS in the presence of or as a result of suspected or proven infection. | - | - | - | Hemodynamic instability and respiratory failure are present.  Life-threatening consequences; urgent intervention indicated | Death |

**A**. Identifying Sepsis

1. Infection is defined as:

A suspected or proven infection caused by any pathogen (by positive culture, tissue stain, or polymerase chain reaction test)

**OR**

A clinical syndrome associated with a high probability of infection. Evidence of infection includes positive findings on clinical exam, imaging, or laboratory tests (i.e. white blood cells in a normally sterile body fluid, perforated viscus, chest radiograph consistent with pneumonia, petechial or purpuric rash, or purpura fulminans).

| **Table 1**  5^th^ and 95^th^ percentile for leukocyte count | |
| --- | --- |
| Age | Leukocyte Count |
| 0 days – 1 week | >34 |
| 1 week to 1 month | >19.5 or <5 |
| 1 month to 1 year | >17.5 or <5 |
| 2 – 5 years | >15.5 or <6 |
| 6 – 12 years | >13.5 or <4.5 |
| 13 – 18 years | >11 or <4.5 |
| Leukocyte Count (10^3^ / mm^3^ = K/Cumm = 10^9^/L) | |

1. SIRS requires the presence of at least two of the following four criteria, one of which must be abnormal temperature (> 38.5°C or <35°C) or leukocyte count (see Table 1).
   1. Core temperature > 38.5°C or <35°C. Note that core temperature must be measured by rectal, bladder, oral or central catheter probe.
   2. Leukocyte count elevated or depressed for age (not secondary to chemotherapy induced leucopenia) or > 10% immature neutrophils (bands) (Table 1)

| **Table 2**  95^th^ percentile values for heart rate in children | |
| --- | --- |
| Age | Beats/minute |
| 0 days – 1 week | 154 |
| 1 week to 1 month | 159 |
| 1 -3 months | 169 |
| 3 – 6 months | 164 |
| 6 - 9 months | 157 |
| 9 – 12 months | 151 |
| 12 – 18 months | 147 |
| 19 – 24 months | 144 |
| 2 – 3 years | 141 |
| 3 – 4 years | 138 |
| 4 – 6 years | 135 |
| 6 – 8 years | 133 |
| 8 – 12 years | 131 |
| 12 – 15 years | 130 |
| 15 – 18 years | 129 |

- 1. Heart Rate (any one of the following):
     1. Tachycardia, defined as: mean heart rate above the 95^th^ percentile for age in the absence of external stimulus, chronic drugs, or painful stimuli (Table 2)

**OR**

- - 1. Otherwise unexplained persistent elevation over a 0.5 to 4 hour time period. Unexplained refers to elevated heart rate not explained by obvious or known cause such as crying, agitated, administration of drugs causing elevated heart rate (atropine), etc.

**OR**

| **Table 3**  10^th^ percentile for heart rate in children < 1 year | |
| --- | --- |
| Age | Beats/minute |
| 0 – 3 months | 123 |
| 3 – 6 months | 120 |
| 6 – 9 months | 114 |
| 9 – 12 months | 109 |

- - 1. Bradycardia for children <1 yr old, defined as: Mean heart rate <10th percentile (Table 3)

**OR**

- - 1. Otherwise unexplained persistent depression of the HR over a 0.5 time period. Unexplained refers to bradycardia not explained by obvious or known cause such as administration of beta-blocking agent, significant hypothermia, known intrinsic dysfunction of the heart’s electrical conduction system (atrioventricular bloc, sinus bradycardia).

| **Table 4**  95^th^ percentile values for respiratory rate in children^51^ | |
| --- | --- |
| Age | Breaths/minute |
| 0 – 3 months | 60 |
| 3 – 6 months | 57 |
| 6 – 9 months | 55 |
| 9 – 12 months | 52 |
| 12- 18 months | 49 |
| 18 – 24 months | 46 |
| 2 – 3 years | 43 |
| 3 – 4 years | 40 |
| 4 – 6 years | 37 |
| 6 - 8 years | 35 |
| 8 – 12 years | 34 |
| 12 – 15 years | 33 |
| 15 – 18 years | 32 |

- 1. Respiratory rate or mechanical ventilation
     1. Mean respiratory rate

above the 95th percentile

for age (Table 4)

**OR**

- - 1. On mechanical ventilation for an acute respiratory process not related to underlying neuromuscular disease or the receipt of general anesthesia.

Mechanical ventilation: Invasive and Non-invasive ventilation.

Invasive ventilation: Mechanical ventilation delivered by positive pressure via endotracheal intubation or laryngeal mask use or a tracheostomy.

Non-invasive ventilation: Mechanical ventilation delivered by bi-level positive airway pressure (BiPAP) through a supra-laryngeal airway by a mask or nasopharyngeal tube. Continuous positive airway pressure (CPAP) throughout the breathing cycle is not considered Non invasive mechanical ventilation.

1. Cardiovascular dysfunction: requires the presence of one of the 3 following criteria:

| Table 5:  <5th percentile for age for systolic BP in children | | |
| --- | --- | --- |
| **Age** | **Boys** | **Girls** |
|  | Systolic BP | Systolic BP |
| 0-7 days | 57 | 57 |
| 8-30 days | 64 | 62 |
| 1-6 months | 72 | 72 |
| 6-12 months | 71 | 70 |
| 1 yr | 74 | 72 |
| 2 yr | 77 | 77 |
| 3 yr | 73 | 72 |
| 4 yr | 71 | 69 |
| 5 yr | 76 | 77 |
| 6 yr | 80 | 78 |
| 7 yr | 81 | 79 |
| 8 yr | 82 | 81 |
| 9 yr | 84 | 82 |
| 10 yr | 85 | 84 |
| 11 yr | 85 | 86 |
| 12 yr | 88 | 89 |
| 13 yr | 87 | 87 |
| 14 yr | 89 | 88 |
| 15 yr | 92 | 89 |
| 16 yr | 94 | 91 |
| 17 yr | 98 | 92 |

1. Decrease in systolic BP (hypotension)

<5th percentile for age (Table 5).

Note that 2 low measurements within

one hour are required.

**OR**

1. Need for vasoactive drug to maintain

BP in normal range (dopamine

>5 mcg/kg/min or any dose of dobutamine,

epinephrine, norepinephrine,

vasopressin/terlipressin,

phenylephrine or milrinone)

**OR**

1. Two of the following

- Unexplained metabolic acidosis,

base deficit ≥5.0 mEq/L

- Increased arterial lactate

>2 times upper limit of normal

- Oliguria: urine output

< 1 mL/kg/hour for 4 hours

- Prolonged capillary refill: > 5 secs
- Core to peripheral temperature gap >3°C.

Core temperature is measured by rectal,

bladder, oral or central catheter probe.

Peripheral temperature is measured by

tympanic, toe, or axillary route.
